# Rapid-onset diarrhoea in HIV patient: The importance of suspecting cholera in non-endemic areas

**DOI:** 10.4102/sajid.v39i1.619

**Published:** 2024-05-31

**Authors:** Meisie A. Nkoane, Adegoke O. Adefolalu

**Affiliations:** 1 Department of Family Medicine and Primary Health Care, Faculty of Medicine, Sefako Makgatho University, Pretoria, South Africa; 2 Practice of Medicine Integrated Programme, School of Medicine, Sefako Makgatho Health Sciences University, Pretoria, South Africa

**Keywords:** cholera, HIV infection, *Vibrio cholerae*, diarrhoea and vomiting, non-endemic area

## Abstract

Cholera, a severe diarrhoeal disease caused by *Vibrio cholerae* is typically associated with inadequate potable water supply and poor sanitation. We report cholera disease presentation identified as a suspected case of acute diarrhoea (HIV/AIDS common condition) in a person living with HIV seen in a cholera non-endemic area.

**Contribution:**

We highlight the importance of recognizing cholera in cases of acute diarrhoea, especially among people with HIV, in resource-constraint areas that lack potable water supply.

## Introduction

*Vibrio cholerae*, a gram-negative bacterium, causes cholera and is classified into serogroups. Serogroups O1 and O139, are mainly responsible for cholera epidemics.^1^ It exhibits a comma-shaped morphology on Gram stain and thrives in brackish waters, forming biofilms and interacting with aquatic organisms. Cholera pathogenesis relies on its virulence factors, primarily the cholera toxin (CT) encoded by *ctxAB* genes, which leads to watery diarrhoea by disrupting intestinal cell signalling, causing chloride ion influx, and passive water secretion.^2^ The toxin-coregulated pilus (TCP) aids in intestinal colonisation. *Vibrio cholerae* also possesses outer membrane proteins, proteases, and secreted factors that contribute to disease. The bacterium evades host defences through mechanisms such as altered lipopolysaccharide (LPS) composition, facilitating effective intestinal colonisation.^3^ Cholera epidemiology is marked by cyclic patterns and seasonal fluctuations, predominantly affecting regions with inadequate sanitation and limited access to clean water,^4^ with annual incidence ranging between 21 000 and 143 000 deaths worldwide because of cholera.^5^ Key risk factors include poor hygiene practices, climate, geography, and socioeconomic conditions. In cholera-endemic countries, an outbreak can be seasonal or sporadic and represents a greater than expected number of cases. In a country or region where cholera does not regularly occur, an outbreak is defined by the occurrence of at least one confirmed case of cholera with evidence of local transmission.^6^ Cholera primarily spreads through ingesting contaminated water, whether from sewage, surface water, or unsanitary sources, which can trigger explosive outbreaks even with minimal contamination.^7^ In addition, the consumption of raw or undercooked seafood and produce washed with contaminated water is a significant transmission route. Although less common, direct person-to-person transmission can occur in densely populated areas and asymptomatic carriers may unknowingly contribute to the disease’s persistence.^8^ Direct exposure within households significantly contributes to the transmission of cholera. Those living close to a cholera patient have a high risk of developing the disease. Cholera can also spread through international travel, with infected individuals introducing the bacteria to new area.^9,10,11^ Environmental factors such as temperature and water availability, along with natural disasters such as floods and earthquakes, can further influence cholera distribution and prevalence by disrupting sanitation infrastructure.^7^ Water, sanitation and hygiene (WASH) interventions are frequently employed to control outbreaks.^12^ In regions where cholera is endemic, HIV infection increases individual susceptibility to cholera. However, in non-endemic areas, accurate reporting of cholera morbidity and mortality rates is frequently hindered by the coexistence of other diarrhoeal diseases, which are occasionally misdiagnosed as unrelated to cholera.^7^ Consequently, the definitive diagnosis of cholera is often overlooked in such settings, because of low index of suspicion among healthcare professionals.^1,7^ We describe a case of cholera in an HIV patient who was seen at a semi-urban district hospital located in a cholera non-endemic area before an outbreak of cholera was declared in May 2023.

## Case presentation

A 48-year-old male living with HIV and from a low-cost housing estate in Kanana, Hammanskraal, north of Pretoria, presented to Jubilee District Hospital’s emergency room (ER) with a 3-day history of diarrhoea and vomiting. His HIV had been diagnosed in 2019 at his local clinic where fixed-dose combination antiretroviral therapy (ART) was started. Clinic records indicated his adherence to the treatment plan. The CD4 count at the time of diagnosis in 2019 was 195 cells/mL, with some element of renal impairment. The CD4 cells increased to 427 cells/mL in 2021. The most recent viral load test in February 2022 revealed an undetectable virus level, and his renal function test from 2021 was normal.

His symptoms for his current admission commenced with abdominal cramps, progressing to watery, non-bloody, and non-mucoid diarrhoea, accompanied by mild vomiting. The diarrhoea was exacerbated by eating, with less than 5 episodes in a 24 h period. The patient initially attributed these symptoms to his HIV disease. However, he sought medical attention at the hospital the following day, feeling unwell, weak, and experiencing lower leg cramps,. He was unemployed, residing with his mother, and had no travel history. He had not been in close contact with individuals suffering from diarrhoea. In addition, he was a non-smoker, abstained from alcohol and illicit drugs, and was not in any relationship. The region where the patient resided relied on municipal water tankers for safe drinking water.

## Clinical findings

- The patient appeared weak, BP 108/78 mmHg, pulse 77 beats/min, respiratory rate 16 breaths/min, temperature 35 °C, oxygen saturation 98%, and blood glucose 6.4 mmol/L.
- Dry mucous membranes, normal lung and heart sounds, and non-tender abdomen with no hepatosplenomegaly
- The remainder of the examination was normal.

In the ER, his HIV status was noted, and he was assessed as having acute gastroenteritis and mild dehydration. Investigations ordered were full blood count (FBC), urea and electrolyte (U&E), C-reactive protein (CRP), and stool microscopy. The medical records indicated an absence of stool collection bottles in the ER and a faulty blood gas machine. The treatment ordered included oral loperamide 2 mg with each loose stool, and intravenous normal saline at 125 mL/h. The patient was admitted to the casualty short-stay area for fluid management and observation while awaiting blood results. The intention was to discharge him within 24 h. At this point, the managing team found no suspicion of cholera. On day 2, the patient’s records indicated persistent diarrhoea and no clinical improvement. A decision was made to admit the patient to a medical ward for further management as soon as a bed became available. The admission laboratory blood results are summarised in Table 1 and Table 2. These results revealed a white cell count of 7.34 × 10^9^/L (normal range [NR] 3.92–10.40), haemoglobin of 15.3 g/dL (NR 13.4–17.5), and a platelet count of 440 × 10^9^/L (NR 171–338). His sodium was 126 mmol/L (NR 136–145) and chloride 93 mmol/L (NR 98–107). Renal impairment was evidenced by a urea of 7.2 mmol/L (NR 2.1–7.1), and creatinine of 338 mmol/L (NR 64–104), with a glomerural fitration rate (GFR) of 18 mL/min.

**TABLE 1:** Haematology blood results.

Item	Results	Reference range
White cell count	7.34 10^9^/L	3.92–10.40
Red cell count	4.98 10^12^/L	4.19–5.85
Haemoglobin	15.3 g/dL	13.4–17.5
MCV	92.2 fL	83.1–1001.6
MCH	30.7 pg	27.8–34.8
MCHC	33.3 g/dL	33.0–35.0
Platelet count	440 109/L H	171–388

*Source:* Data obtained from the National Health Laboratory Service (NHLS) and used with permission.

MCV, mean corpuscular volume; MCH, mean corpuscular haemoglobin; MCHC, mean corpuscular haemoglobin concentration.

**TABLE 2:** Chemical pathology results.

Item	Results	Reference range
Sodium	126 L mmol/L	136–145
Potassium	4.8 mml/L	3.5–5.1
Chloride	93 mmol/L	98–107
Bicarbonate	13 L mmol/L	23–29
Anion gap	25 H mmol/L	9–16
Urea	7.2 H mmol/L	2.1–7.1
Creatinine	338 H mmol/L	64–104
eGFR (CKD-EPI formula)	18 mL/min/1.73 m^2^	> 60
C-Reactive protein	22 H mg/L H	˂ 10

*Source*: Data obtained from the National Health Laboratory Service (NHLS) and used with permission.

eGFR, estimated glomerular filtration rate; CKD-EPI, Chronic Kidney Disease Epidemiology Collaboration.

The patient’s condition remained unchanged, and a stool specimen was sent to the laboratory for cholera testing on day 2 of hospitalisation. This was prompted by an awareness that increasing numbers of patients were presenting to the hospital with diarrhoeal diseases. Also, the clinical manager had alerted the medical team of the possible outbreak of cholera. Intravenous treatment with Ringer’s Lactate continued, loperamide was stopped and ciprofloxacin 500 mg twice daily was initiated. However, his condition deteriorated in the early hours of day 5 in the hospital. His blood pressure dropped to 71/32 mmHg, with a bradycardia of 54 beats/min. Resuscitation was unsuccessful and the patient unfortunately died a few hours later. The stool results later on confirmed *V. cholerae* on day 5 (see Table 3).

**TABLE 3:** Microbiologic stool results.

Item	Observation
**Stool analysis**	
Appearance	Watery
Wet preparation	Not observed
Leucocytes	Not observed
Erythrocytes	Not observed
Epithelial cells	Not observed
Yeasts	Not observed
Parasite(s)	Not observed
**Bacterial culture**	
Pathogen(s) isolated: 1	*Vibrio cholerae* (VIBCH) This is a notifiable condition according to the National Health Act, Act 61 of 2003.

*Source:* Data obtained from the National Health Laboratory Service (NHLS) and used with permission.

## Discussion

The patient was initially assessed as a person living with HIV, presenting with acute gastroenteritis and dehydration. The treatment plan included admission, blood tests (FBC, U&E, CRP), stool microscopy, culture and sensitivity (MC&S). However, there were initial delays with stool specimen collection and processing. Stool analysis ultimately confirmed the presence of *V. cholerae* but initial submission was delayed. Laboratory tests showed hyponatraemia and acute kidney injury, likely due to dehydration. HIV-associated nephropathy (HIVAN) may have also played a role. Nevertheless, the patient’s management was not optimal. It is difficult to ascertain whether the outcome would have been better if his presentation was because of another cause of diarrhoea. The amount of fluid that was lost and the rapidity with which it needed to be replaced was underestimated. The patient received too little intravenous fluid during the initial phase of rehydration. The apparent delayed diagnosis because of the lack of awareness regarding cholera in the community also played a critical role in this case, the attending doctors and nurses assumed they were dealing with ordinary diarrhoea.^7^ In week 7 of 2024, the WHO regional cholera update reported 3216 new cases in 10 countries (Burundi, Cameroon, Comoros, Ethiopia, Kenya, Malawi, Mozambique, United Republic of Tanzania, Zambia, and Zimbabwe).^12^

Our case report demonstrates why a high index of suspicion for cholera is needed in settings where there is history of water and sanitation problems, irrespective of the region’s classification as cholera non-endemic. Prompt intervention and close monitoring are essential to manage renal and electrolyt abnormalities. Timely diagnosis, proper rehydration, and medical community awareness are crucial for better outcomes in cholera patients. Undoubtedly access to clean water and sanitation is essential for preventing cholera transmission and other waterborne diseases at the community level, in addition to maintaining a high level of suspicion within the health facilities.

## Conclusion

We presented a case of cholera in a patient with well controlled HIV who was managed for acute diarrhoea, a common condition in people living with HIV. Cholera was not suspected because the patient lived within the peri-urban precincts of Pretoria, a cholera non-endemic area. Since the cholera outbreak was recognised, the National Department of Health pooled together state and non-state actors at the national, provincial, district and local levels for joint efforts on cholera prevention and control. These include prioritising cross-border collaboration, investing in WASH, as well as education on hygiene practices. This case report provides insights into the importance of early recognition of cholera and prompt initiation of appropriate management, even in cholera non-endemic regions. We therefore recommend a high index of suspicion for cholera in patients presenting with acute diarrhoea and dehydration.

## References

[1] Gladney LM, Griswold T, Turnsek M, et al. Characterization of a nonagglutinating toxigenic *Vibrio cholerae* isolate. Microbiol Spectr. 2023;11(3):e0018223. 10.1128/spectrum.00182-23 37195209 PMC10269536

[2] Pant A, Das B, Bhadra RK. CTX phage of Vibrio cholerae: Genomics and applications. Vaccine. 2020;38:A7–A12. 10.1016/j.vaccine.2019.06.034 31272871

[3] Nelson EJ, Harris JB, Morris JG Jr, Calderwood SB, Camilli A. Cholera transmission: The host, pathogen, and bacteriophage dynamic. Nat Rev Microbiol. 2009;7(10):693–702. 10.1038/nrmicro2204 19756008 PMC3842031

[4] Cholera is endangering children globally [homepage on the Internet]. UNICEF; 2023 [cited 2024 Mar 09]. Available from: https://www.unicef.org/stories/cholera-is-endangering-children-globally

[5] Cholera [homepage on the Internet]. World Health Organization (WHO); 2021 [cited 2024 Mar 09]. Available from: https://www.who.int/news-room/facts-in-pictures/detail/cholera

[6] Deen J, Mengel MA, Clemens JD. Epidemiology of cholera. Vaccine. 2020;38:A31–A40. 10.1016/j.vaccine.2019.07.078 31395455

[7] Valcin CL, Severe K, Riche CT, et al. Predictors of disease severity in patients admitted to a cholera treatment center in urban Haiti. Am J Trop Med Hyg. 2013;89(4):625–632. 10.4269/ajtmh.13-0170 24106188 PMC3795091

[8] Connor BA, Dawood R, Riddle MS, Hamer DH. Cholera in travellers: A systematic review. J Travel Med. 2019;26(8):taz085. 10.1093/jtm/taz085 PMC6927393 31804684

[9] Disease A-Z Index. Cholera diagnosis and case management 2023 [homepage on the Internet]. National Institute for Communicable Diseases. [cited 2024 Mar 09]. Available from: https://www.nicd.ac.za/diseases-a-z-index/cholera/

[10] Von Seidlein L, Wang X-Y, Macuamule A, et al. Is HIV infection associated with an increased risk for cholera? Findings from a case–control study in Mozambique. Trop Med Int Health. 2008;13:683–688. 10.1111/j.1365-3156.2008.02051.x 18331384

[11] Taylor DL, Kahawita TM, Cairncross S, Ensink JH. The impact of water, sanitation and hygiene interventions to control cholera: A systematic review. PLoS One. 2015 Aug 18;10(8):e0135676. 10.1371/journal.pone.0135676 26284367 PMC4540465

[12] Cholera in the WHO African Region – Weekly regional cholera bulletin [homepage on the Internet]. 2024 [cited 2024 Mar 15]. Available from: https://www.afro.who.int/health-topics/disease-outbreaks/cholera-who-african-region

